# Deep learning-based synthetic dose-weighted LET map generation for intensity modulated proton therapy

**DOI:** 10.1088/1361-6560/ad154b

**Published:** 2024-01-05

**Authors:** Yuan Gao, Chih-Wei Chang, Shaoyan Pan, Junbo Peng, Chaoqiong Ma, Pretesh Patel, Justin Roper, Jun Zhou, Xiaofeng Yang

**Affiliations:** 1 Department of Radiation Oncology and Winship Cancer Institute, Emory University, Atlanta, GA, United States of America; 2 Department of Biomedical Informatics, Emory University, Atlanta, GA, United States of America; 3 Department of Nuclear & Radiological Engineering and Medical Physics, Georgia Institute of Technology, Atlanta, GA, United States of America

**Keywords:** LET, cycleGAN, deep learning, dose, synthetic

## Abstract

The advantage of proton therapy as compared to photon therapy stems from the Bragg peak effect, which allows protons to deposit most of their energy directly at the tumor while sparing healthy tissue. However, even with such benefits, proton therapy does present certain challenges. The biological effectiveness differences between protons and photons are not fully incorporated into clinical treatment planning processes. In current clinical practice, the relative biological effectiveness (RBE) between protons and photons is set as constant 1.1. Numerous studies have suggested that the RBE of protons can exhibit significant variability. Given these findings, there is a substantial interest in refining proton therapy treatment planning to better account for the variable RBE. Dose-average linear energy transfer (LET_d_) is a key physical parameter for evaluating the RBE of proton therapy and aids in optimizing proton treatment plans. Calculating precise LET_d_ distributions necessitates the use of intricate physical models and the execution of specialized Monte-Carlo simulation software, which is a computationally intensive and time-consuming progress. In response to these challenges, we propose a deep learning based framework designed to predict the LET_d_ distribution map using the dose distribution map. This approach aims to simplify the process and increase the speed of LET_d_ map generation in clinical settings. The proposed CycleGAN model has demonstrated superior performance over other GAN-based models. The mean absolute error (MAE), peak signal-to-noise ratio and normalized cross correlation of the LET_d_ maps generated by the proposed method are 0.096 ± 0.019 keV *μ*m^−1^, 24.203 ± 2.683 dB, and 0.997 ± 0.002, respectively. The MAE of the proposed method in the clinical target volume, bladder, and rectum are 0.193 ± 0.103, 0.277 ± 0.112, and 0.211 ± 0.086 keV *μ*m^−1^, respectively. The proposed framework has demonstrated the feasibility of generating synthetic LET_d_ maps from dose maps and has the potential to improve proton therapy planning by providing accurate LET_d_ information.

## 1. Introduction

Proton therapy has an advantage over conventional photon therapy because of the proton beam Bragg peak effect (Goitein 1985, Baumann *et al* 2020). The Bragg peak effect allows a proton to deposit most of its energy in the tumor while sparing normal tissues because there is essentially no exit dose beyond the Bragg Peak. Proton therapy offers advantages, but it does come with uncertainty due to different dose effects compared to conventional photon radiotherapy. Bragg Peak positioning is strongly dependent on the accurate estimation of the proton range, which can be complicated by variations in tissue composition and density within the patient’s body, introducing uncertainties that are not typically encountered with photon therapy (Paganetti 2012). Perhaps more importantly, the difference in biological effectiveness between protons and photons is not fully modeled in the current commercial treatment planning systems (TPS) (McMahon *et al* 2018).

In order to leverage the comprehensive experience gained from photon treatments, prescriptions for proton therapy are typically created based on the physical dose, multiplied by a factor. This factor is used to account for the difference in biological effects that occur when delivering the same physical dose with photons as compared to protons. Theoretically, this kind of difference between protons and photons is quantified by relative biological effectiveness (RBE), which is the ratio of doses from different radiation types to reach the same biological effects. In current clinical practice, the planning and delivery of proton therapy treatments typically operate under the assumption that the RBE between protons and photons is a constant value of 1.1 (Paganetti 2014). This value is an average of RBE values measured *in vivo*, ignoring the properties of protons that vary by depth in tissue. More specifically, the uncertainty of this constant approximation increases at the distal end of the proton range. Numerous research studies have examined whether these RBE variations are relevant in clinical practice. These studies suggest that the RBE of proton therapy can vary significantly, ranging from 1 to potentially more than 2 (Guan *et al* 2015a, Peeler *et al* 2016, Yang *et al* 2022). Given these findings, there is a substantial interest in refining proton therapy treatment planning to better account for this variability in RBE.

For a specific type of radiation, RBE is strongly influenced by the linear energy transfer (LET) of the radiation, which is the average amount of energy loss of particles per unit track length in tissue. Consequently, there is clinical interest to reconstruct three-dimensional LET distributions, in addition to the physical dose distributions, for more accurate RBE calculation and subsequent more precise proton treatment. Specifically, this approach could potentially aid in identifying regions of high LET, where substantial RBE variations are anticipated, or even assist in estimating three-dimensional RBE distributions. In clinical practice, dose-averaging LET (LET_d_) is used for relating RBE value to dosimetry parameters (Paganetti 2014). However, the LET_d_ is not a physical quantity that can be easily measured. Thus, this value is typically calculated using analytical methods (Wilkens and Oelfke 2003, 2004, Hirayama *et al* 2018), or Monte Carlo (MC) simulations (Kraft *et al* 1992, Guan *et al* 2015b, Bertolet *et al* 2019). The MC technique is generally considered the most accurate method for simulating complex radiation transport in heterogeneous medium (Chang *et al* 2020). However, implementing the MC simulation demands considerable effort, and the simulation process itself can be quite time-consuming.

Knowledge-based systems, tracing their origins back to the 1980s, were established to aid in the design of radiation treatment plans. Central to these systems were expert-driven methodologies, which encapsulated the extensive knowledge and experience of clinicians into well-defined rules and algorithms (Ge and Wu 2019). Fast-forwarding to the present, ‘knowledge-based planning’ (KBP) predominantly refers to data-driven approaches in the domain of treatment planning. Capitalizing on historical data and cutting-edge computational strategies, these methodologies facilitate and elevate the planning procedures. One of the key strengths of KBP models lies in their ability to rapidly churn out plan predictions based on specific feature sets, enabling an immediate appraisal of variances between different modalities. Such prompt assessments negate the typical wait-time associated with crafting exhaustive treatment plans, which often spans several days. With KBP in their arsenal, clinical teams can strategically direct their energies towards the optimal modality, sidestepping the tedious chore of designing myriad treatment plans before finalizing a strategy. This surge in clinical efficacy and uniformity can bestow significant benefits, especially in specialized areas like proton therapy. Intriguingly, to date, KBP methodologies haven’t been explored in the context of LET_d_.

To ensure the provision of LET_d_ distributions for future treatment planning optimization and to bypass the challenges associated with executing MC simulations and provide a potential for KBP approaches, we put forth a novel framework. This design not only addresses the current challenges but also paves the way for potential integration with KBP approaches. This strategy uses a deep learning (DL) technique to create synthetic LET_d_ distribution maps from dose distribution maps. Cycle generative adversarial network (CycleGAN) has proven its superiority over other DL models in image synthesis (Zhu *et al* 2017, Harms *et al* 2019, Lei *et al* 2019, Liu *et al* 2021), and we developed a supervised CycleGAN to synthesize LET_d_ maps based on dose information. This study provides an in-depth explanation of the network structure and the hyperparameters utilized. The purpose of the proposed work is to provide a feasible solution to deliver highly accurate LET_d_ information to support medical decision-making in clinic without implementing LET-based MC simulations. This work, to our knowledge, is the first of its kind to demonstrate the capability of providing LET_d_ information for prostate cancer patients undergoing stereotactic body radiation therapy (SBRT) treatment.

## 2. Materials and methods

Our proposed framework for generating synthetic LET_d_ maps, derived from dose maps, encompasses two stages: a training stage and a test stage. For this project, we utilized data from 50 patients; We employed a 5-fold cross-validation approach in our methodology. In this process, our patient dataset was evenly divided into five subsets. During each iteration of the validation, a single subset of patients was selected as the test dataset, with the remaining four subsets used for training. This procedure was repeated five times, each time with a different subset serving as the test dataset, thereby ensuring that each patient’s data was included in the test dataset exactly once. Emory IRB review board approval was obtained, and informed consent was not required for this Health Insurance Portability and Accountability Act (HIPAA) compliant retrospective analysis. Three DL models were compared in our study: the pixel-to-pixel GAN, the Wasserstein CycleGAN, and our proposed CycleGAN. These DL models were trained using 2D transverse slices and were subsequently evaluated based on quantitative parameters.

### 2.1. Proposed cycleGAN based framework

**Figure 1.**
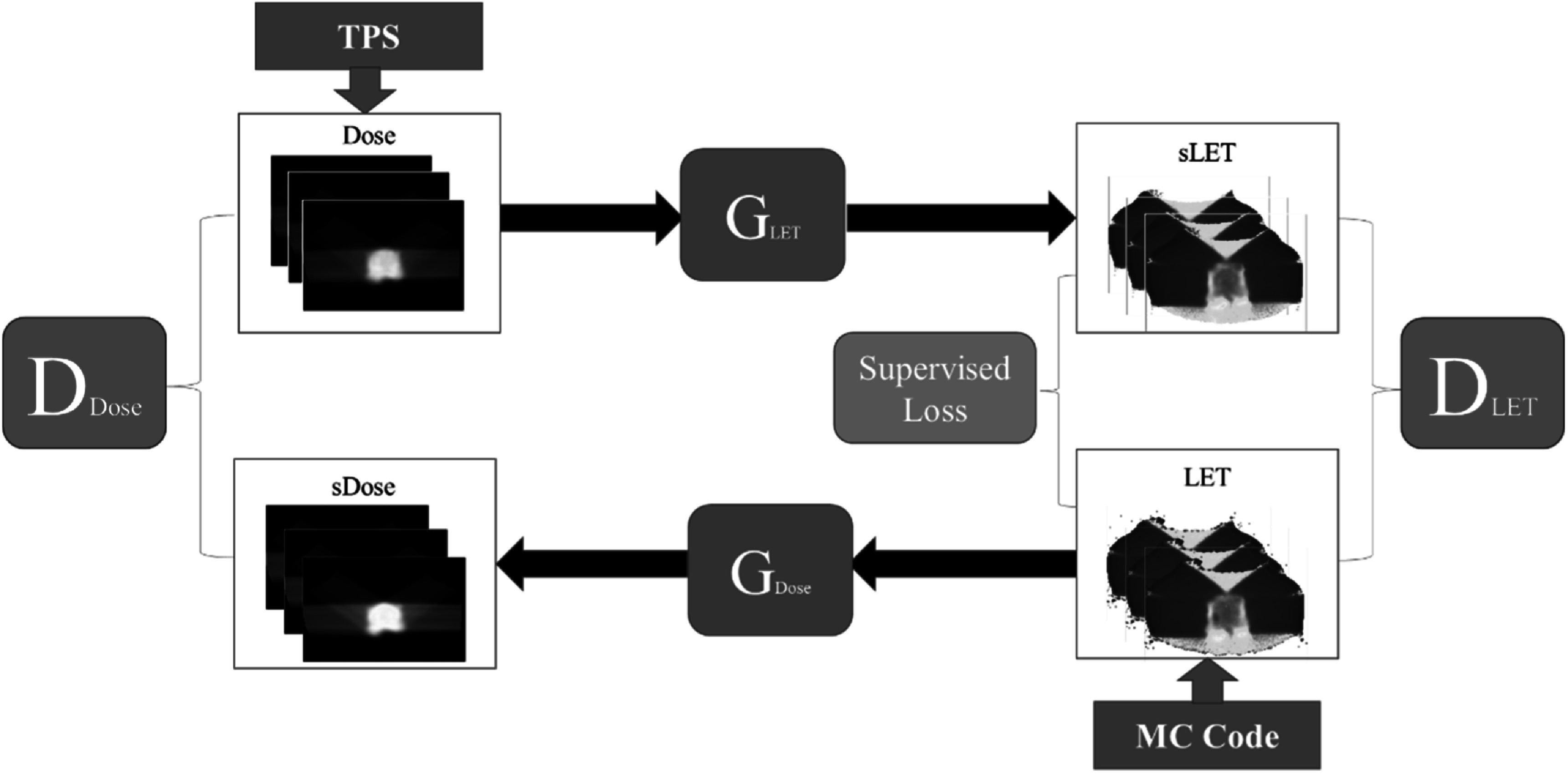
Proposed CycleGAN based synthetic LET_d_ map generation framework. D_Dose_ represents the dose image discriminator, D_LET_ is the LET_d_ image discriminator, G_LET_ is the generator that generates LET_d_ image from dose image, G_Dose_ is the dose generator from LET_d_ image, Dose in blue square means the original dose image, LET in blue square means the ground truth LET_d_ image, sDose in red square means the synthetic dose image, sLET in red square means the synthetic LET_d_ images, Supervised Loss is the added compound loss function compare to traditional cycleGAN. The ground truth dose image goes into G_LET_ to generate sLET, while the ground truth LET_d_ enters G_Dose_ to generate sDose, thereby completing the cycle. The sLET and ground truth LET_d_ enters the D_LET,_ while the sDose and ground truth Dose enters the D_Dose_.

The detailed framework is shown in figure 1. The dose distribution map for the patient was derived from the TPS of RayStation 10B (RaySearch Lab., Stockholm, Sweden). Conversely, the LET_d_ distribution map was produced using a Monte Carlo (MC) engine in RayStation 12 A (RaySearch Lab., Stockholm, Sweden), which has been validated against FLUKA simulation reference.

#### 2.1.1. Proposed cycleGAN architecture

Traditional adversarial network methods, such as generative adversarial networks (GANs), are founded on two sub-networks: a generator and a discriminator, operating in opposing directions. Given two training datasets—for instance, a dose map and a LET_d_ map—an initial mapping is learned to generate an image that resembles an LET_d_ map from a dose map image, referred to as a synthetic LET_d_ map image in this study. The generator’s task is to create a synthetic LET_d_ map image from the dose map image that is convincing enough to deceive the discriminator into identifying it as a genuine LET_d_ map image. Conversely, the discriminator’s training objective is to minimize the judgment error within its network and to amplify its capacity to discern a genuine LET_d_ map image from a synthetic LET_d_ map image. The generator and discriminator networks compete against each other during the training process. This competition fosters the improvement of each network’s capabilities, leading to a more accurate synthetic LET_d_ map in this study (Goodfellow *et al* 2020).

**Figure 2.**
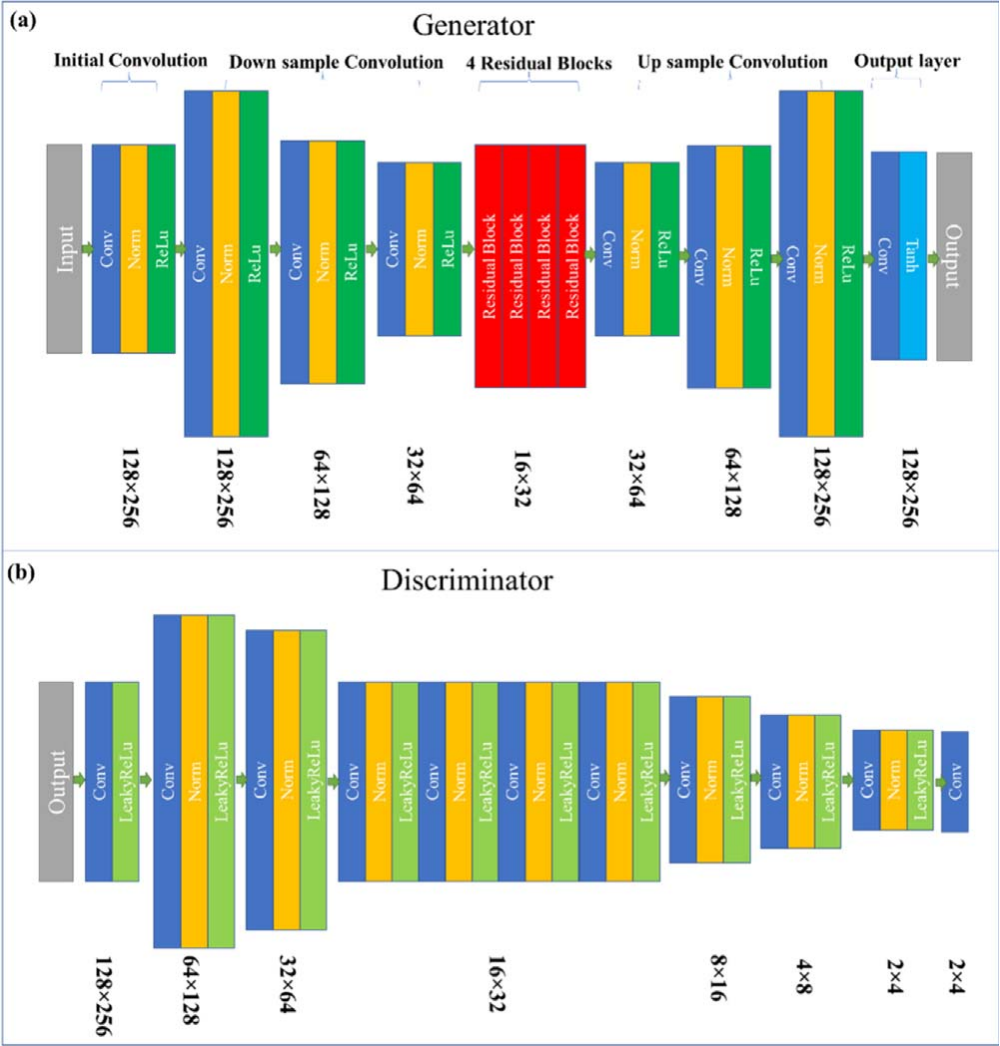
Proposed cycleGAN detail structure of generator (a) and discriminator (b).

The CycleGAN, developed by Zhu *et al* (2017) is a novel approach for unpaired image-to-image translation based on the GAN framework. Its superiority over traditional DL networks in generating synthetic images has been affirmed by various research studies (Lei *et al* 2019, Harms *et al* 2020, Brou Boni *et al* 2021). The proposed CycleGAN consists of two sub-networks: two U-net based generators and two discriminators. Their architecture detail information are shown in figures 2(a) and (b). The structure of the generators and discriminators was specifically designed and tailored to accommodate the unique characteristics of these datasets and the intended training objectives. In this study, the two generators within the CycleGAN are trained with the aim of creating synthetic LETd map images based on authentic dose map images and generating synthetic dose map images grounded on authentic LET_d_ map images. Simultaneously, the two discriminators within the CycleGAN are trained with the goal of distinguishing between the true and synthetic LET_d_ map and dose map images. The CycleGAN model enforces an inverse transformation, thereby doubly constraining the model. This additional constraint can potentially enhance the accuracy of the output image (Zhu *et al* 2017).

**Figure 3.**
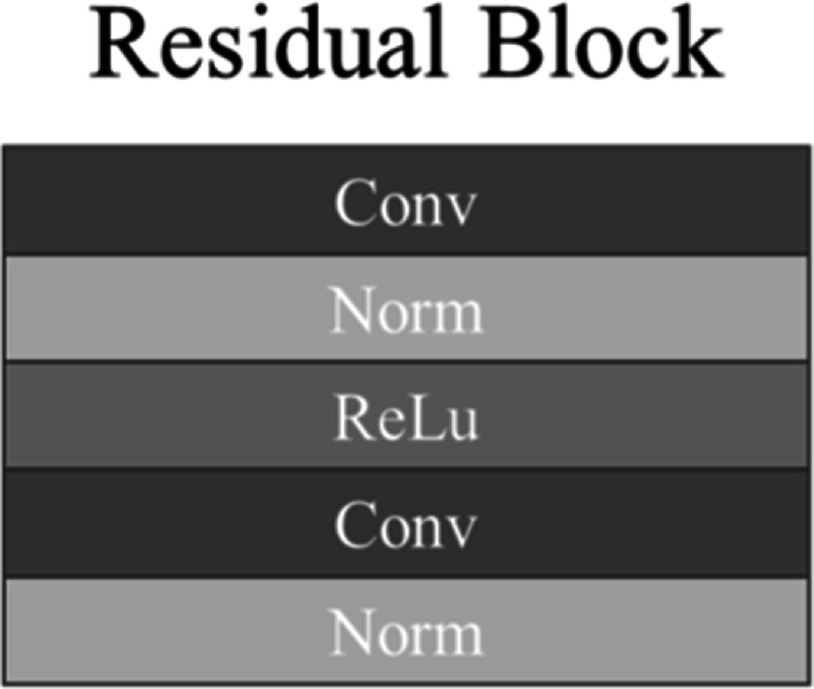
Proposed cycleGAN residual block detail structure.

Convolutional neural networks (CNNs) that incorporate residual blocks have demonstrated impressive results in image-to-image translation tasks (He *et al* 2016), particularly when the source and target images share substantial similarity. This is akin to the relationship between dose and LET_d_ map images. Detailed information of residual block in generator is shown in figure 3. Each residual block consists of a residual connection and multiple hidden layers. Although the residual block doesn’t alter the size of the feature map, it facilitates network optimization and enhances performance in image-to-image translation tasks.

#### 2.1.2. Compound loss function

CycleGAN generator loss function can be divided into three parts, an adversarial loss also called GAN loss, cycle consistency loss and identity loss (Zhu *et al* 2017). The adversarial loss function, which relies on the output of two discriminators, applied to both the dose-to-LET_d_ (G_LET_) generator and the LET_d_-to-dose generator (G_Dose_), and they are shown in equations (1) and (2)

\begin{eqnarray*}{{\mathscr{L}}}_{\mathrm{adv},{D}_{\mathrm{LET}}}\left({D}_{\mathrm{LET}},{I}_{\mathrm{Dose}},{I}_{\mathrm{noise}}\right)=-{D}_{\mathrm{LET}}({G}_{\mathrm{LET}}\left({I}_{\mathrm{Dose}}\right))\,\end{eqnarray*}

\begin{eqnarray*}{{\mathscr{L}}}_{{\mathrm{adv},D}_{\mathrm{DOSE}}}\left({D}_{\mathrm{Dose}},{I}_{\mathrm{LET}},{I}_{\mathrm{noise}}\right)=-{D}_{\mathrm{Dose}}({G}_{\mathrm{Dose}}\left({I}_{\mathrm{LET}}\right)).\end{eqnarray*}

The adversarial loss function alone is not sufficient for synthetic image generation, and it leaves the model under-constrained. It enforces that the generated output be of the target domain but does not enforce that the input and output are recognizably the same, while the cycle consistency loss addresses this issue. Two cycle consistency loss function are shown in equations (3) and (4). Where ${\unicode{x02016}\unicode{x02016}}_{1}$ means the L1 norm operator

\begin{eqnarray*}{{\mathscr{L}}}_{\mathrm{cyc},\mathrm{Dose}-\mathrm{LET}-\mathrm{Dose}}\left({G}_{\mathrm{LET}},{G}_{\mathrm{Dose}},{I}_{\mathrm{LET}},{I}_{\mathrm{Dose}}\right)={\unicode{x02016}{{G}_{\mathrm{Dose}}(G}_{\mathrm{LET}}\left({I}_{\mathrm{Dose}}\right)),{I}_{\mathrm{Dose}}\unicode{x02016}}_{1}\end{eqnarray*}

\begin{eqnarray*}{{\mathscr{L}}}_{\mathrm{cyc},\mathrm{LET}-\mathrm{Dose}-\mathrm{LET}}\left({G}_{\mathrm{LET}},{G}_{\mathrm{Dose}},{I}_{\mathrm{LET}},{I}_{\mathrm{Dose}}\right)={\unicode{x02016}{{G}_{\mathrm{LET}}(G}_{\mathrm{Dose}}\left({I}_{\mathrm{LET}}\right)),{I}_{\mathrm{LET}}\unicode{x02016}}_{1}.\end{eqnarray*}

The third part of loss function is identity loss. The purpose of this additional loss function is to preserve color composition the edges of synthetic LET maps from ground truth. Without the constraint of this identity loss, the generator G_LET_ and G_Dose_ may change the tint of input images when it is not necessary. The identity loss function is shown in equations (5) and (6)

\begin{eqnarray*}{{\mathscr{L}}}_{\mathrm{identity},\mathrm{Dose}}({G}_{\mathrm{LET}},{I}_{\mathrm{LET}})={\unicode{x02016}{G}_{\mathrm{LET}}\left({I}_{\mathrm{LET}}\right),{I}_{\mathrm{LET}}\unicode{x02016}}_{1}\end{eqnarray*}

\begin{eqnarray*}{{\mathscr{L}}}_{\mathrm{identity},\mathrm{LET}}({G}_{\mathrm{Dose}},{I}_{\mathrm{Dose}})={\unicode{x02016}{G}_{\mathrm{Dose}}\left({I}_{\mathrm{Dose}}\right),{I}_{\mathrm{Dose}}\unicode{x02016}}_{1}.\end{eqnarray*}

Traditional CycleGANs are designed for unpaired image-to-image translation. However, in this work, we have paired images, leading us to propose a supervised CycleGAN (referred to as the proposed Cycle GAN). The architecture of the proposed CycleGAN network is presented in figure 1, which includes an additional supervised loss function incorporated into the generator’s loss function. This added supervised loss function in this study is outlined in equations (7) and (8), where ILET is the true LETd map image, IDose is the true dose map image, and the function ${\unicode{x02016}\unicode{x02016}}_{2}$ is the L2 norm operator. The total generator loss function is shown in equation (11), where ${{\mathscr{L}}}_{\mathrm{adv}},$ ${{\mathscr{L}}}_{{cyc}},$ ${{\mathscr{L}}}_{\mathrm{identity}}$ are the summation of adversarial, cycle consistency and identity loss function, where $\lambda ,$ $\beta ,$ ${\mathrm{\gamma }}$ and ${\mathrm{\delta }}$ are the weight parameters.

\begin{eqnarray*}{{\mathscr{L}}}_{\mathrm{Dose}-\mathrm{LET}}\left({G}_{\mathrm{LET}},{I}_{\mathrm{Dose}},{I}_{\mathrm{LET}}\right)={\unicode{x02016}{G}_{\mathrm{LET}}\left({I}_{\mathrm{Dose}}\right)-{I}_{\mathrm{LET}}\unicode{x02016}}_{2}\end{eqnarray*}

\begin{eqnarray*}{{\mathscr{L}}}_{\mathrm{LET}-\mathrm{Dose}}\left({G}_{\mathrm{Dose}},{I}_{\mathrm{Dose}},{I}_{\mathrm{LET}}\right)={\unicode{x02016}{G}_{\mathrm{Dose}}\left({I}_{\mathrm{LET}}\right)-{I}_{\mathrm{Dose}}\unicode{x02016}}_{2}\end{eqnarray*}

\begin{eqnarray*}{{\mathscr{L}}}_{{\mathrm{G}},\mathrm{total}}={{\mathscr{L}}}_{\mathrm{adv}}+\lambda {{\mathscr{L}}}_{\mathrm{cyc}}+\beta {{\mathscr{L}}}_{\mathrm{identity}}+\gamma {{\mathscr{L}}}_{\mathrm{Dose}-\mathrm{LET}}+\delta {{\mathscr{L}}}_{\mathrm{LET}-\mathrm{Dose}}.\end{eqnarray*}

The CycleGAN discriminator loss consists of two parts, the fake loss and real loss, and they are shown in equations (10) and (11). The total discriminator loss function is shown in equation (12). Where ${I}_{\mathrm{noise}}$ represents a generated noise image where each voxel possesses a random value between 0 and 1, $\lambda $ is the weight parameters which is set as 0.5 in this work

\begin{eqnarray*}{{\mathscr{L}}}_{\mathrm{Dose}-\mathrm{LET}}\left(D,{I}_{\mathrm{LET}}\right)={D}_{\mathrm{LET}}\left({G}_{\mathrm{LET}}\left({I}_{\mathrm{Dose}}\right)\right)-{D}_{\mathrm{LET}}({I}_{\mathrm{LET}}).\end{eqnarray*}

\begin{eqnarray*}{{\mathscr{L}}}_{\mathrm{LET}-\mathrm{Dose}}\left(D,{I}_{\mathrm{Dose}}\right)={D}_{\mathrm{Dose}}\left({G}_{\mathrm{Dose}}\left({I}_{\mathrm{LET}}\right)\right)-{D}_{\mathrm{Dose}}({I}_{\mathrm{Dose}})\end{eqnarray*}

\begin{eqnarray*}{{\mathscr{L}}}_{D}\left(D,{I}_{\mathrm{LET}},{I}_{\mathrm{noise}}\right)={{\mathscr{L}}}_{\mathrm{Dose}-\mathrm{LET}}\left(D,{I}_{\mathrm{LET}}\right)+{{\mathscr{L}}}_{\mathrm{LET}-\mathrm{Dose}}\left(D,{I}_{\mathrm{Dose}}\right).\end{eqnarray*}

### 2.2. Data acquisition and preprocess

The data set consisted of 50 patients diagnosed with early-stage prostate cancer, all of whom received proton SBRT at the Emory Proton Therapy Center. Out of these, 41 patients were designated as the training dataset, while the remaining 9 patients were used as the application dataset. All the patients received a total dosage of 36.25 Gy_RBE_, administered over 5 fractions. Each treatment plan incorporated four beams: two horizontal opposed beams and two anterior oblique beams set at +/− 45-degree angle from the horizontal level. The two horizontally opposed beams accounted for 70% of the total doses. All treatment plans were optimized using the RayStation treatment planning system V10B (Raysearch Lab, Stockholm, Sweden). As per clinical practice, we planned on the clinical target volume (CTV) with robust optimization accounting for an uncertainty of ±3.5% in range and 5 mm (3 mm in posterior) in 6 orthogonal directions for setup. Single field optimization (SFO) strategies were mostly utilized in the treatment planning process.

**Figure A1.**
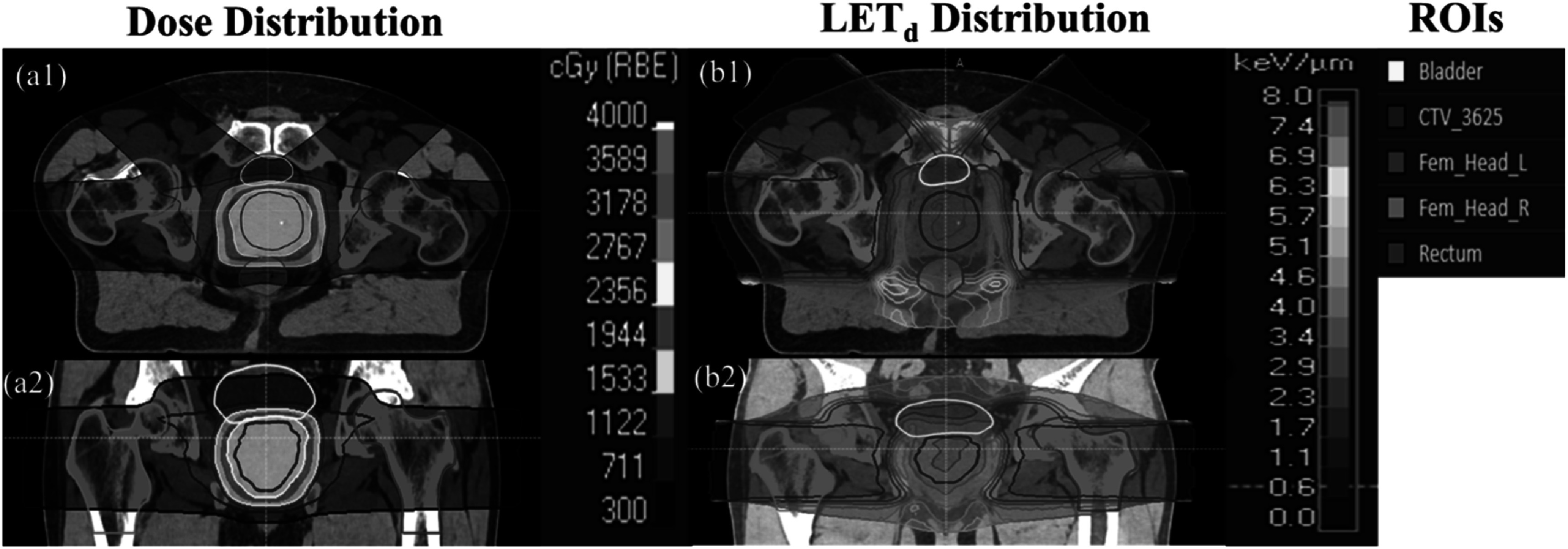
Patient dose maps in transversal (a1) and coronal (a2) views generated from RayStation. The corresponding patient LET_d_ maps in transversal (b1) and coronal (b2) views generated from RayStation. The regions-of-interest (ROIs) shown in the figure include CTV, Rectum, Bladder, left femur head (Fem_Head_L) and right femur head (Fem_Head_R).

**Figure A2.**
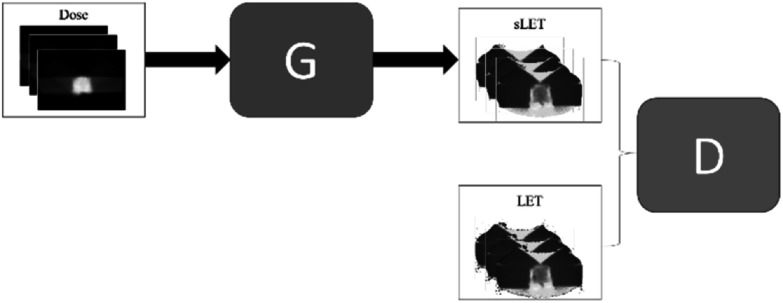
Pix2PixGAN structure. Dose in blue square means the original dose image, G is the generator, D is the discriminator, LET in blue square means the ground truth LET_d_ image, sLET in red square means the synthetic LET_d_ images.

The total dose maps were produced with Monte Carlo dose engine implemented in RayStation V10B. All treatment plans shared the same dose grid, with a resolution of 0.3 cm × 0.3 cm × 0.3 cm. The dose map with patient CT are shown in figures A1(a1)–(a2). The LET_d_ is defined as equation (13), where ${{\mathrm{\varnothing }}}_{E}\left(x\right)$ is the proton fluence at specific distance *x*, and *S*(*E*) is the stopping power at specific energy. The LET_d_ map was calculated with RayStation V12A MC code engine (RaySearch Lab., Stockholm, Sweden), which has been verified with experiment and FLUKA (AB 2021). The calculated LET_d_ maps with patient CT were shown in figures A2(b1)–(b2). The LET_d_ has the same voxel size with dose map, which is 0.3 cm, and the LET_d_ is shown in the unit of 1 $\mathrm{KeV}\,\mu {{\mathrm{m}}}^{-1}.$ A dose threshold (50 cGy_RBE_) has been added for the calculated LET_d_. A series of Python scripts was used to extract the dose and LET_d_ map from RayStaion into a three-dimensional matrix. Then the dose and LET_d_ maps were exported to MATLAB R2021b (Mathworks, Natick, MA). Given that each patient has a unique CTV, both the dose and LET_d_ maps exhibit varying image sizes, despite sharing the same spatial resolution. The DL training necessitates uniform image sizes within the training dataset. Therefore, all exported images were padded with zeros to achieve a consistent size of 128 × 256 × 64

\begin{eqnarray*}{\mathrm{LET}}_{d}=\frac{{\int }_{0}^{{\mathrm{\infty }}}{\varnothing }_{E}\left(x\right){S}^{2}\left(E\right){dE}}{{\int }_{0}^{{\mathrm{\infty }}}{\varnothing }_{E}\left(x\right)S\left(E\right){dE}}.\end{eqnarray*}

### 2.3. Reference models

To assess the proposed framework, three benchmark models were employed: a CNN, the pixel-to-pixel GAN (often referred to as Pix2PixGAN), and the Wasserstein GAN. A convolutional neural network (CNN) is a DL algorithm primarily designed for tasks related to processing data with a grid-like topology, such as images. It is one of the foundational architectures behind many advancements in the domain of computer vision (O’Shea and Nash 2015). The implementation of the CNN in our research builds upon our prior publications. For a detailed insight into its implementation, readers are referred to our preceding works (Chang *et al* 2022a, 2022b, Chih-Wei Chang *et al* 2022, Gao *et al* 2022, Chang *et al* 2023, Gao *et al* 2023).

The primary distinction between CycleGAN and Pix2PixGAN (Isola *et al* 2017) is their respective generative mechanisms and. Specifically, CycleGAN operates in an unsupervised manner and employs a dual generative process, while Pix2PixGAN, on the other hand, operates in a supervised manner, using a paired dataset, and utilizes a single generative process. In our research, we leveraged the Pix2PixGAN for the synthesis of the LET_d_ map. A visual representation of the Pix2PixGAN architecture is depicted in figure A2, with detailed structures of the U-net generator (G) and discriminator (D) shown in figures 2(a) and (b) respectively. Within the scope of our study, the Pix2PixGAN functions as a generative model, establishing a transformation from *x* (dose map image) to *y* (LET_d_ map image).

The Wasserstein CycleGAN, an extension of the classic CycleGAN, was introduced by Martin *et al* It integrates the Wasserstein distance metric, as described by Arjovsky *et al* (2017), to bolster training stability and diminish the potential for mode collapse. While the architecture of the Wasserstein CycleGAN mirrors that of the traditional CycleGAN, depicted in figure 1(b), the specifics of the generator and discriminator can be observed in figures 2(a) and (b), respectively. Additionally, the design of the residual block is illustrated in figure 3.

### 2.4. Implementation and evaluation

The dose and LET_d_ map images were fed into the network as 128 × 256 2D slices. Given that each patient has 64 slices and considering the dataset of 41 patients, there are a total of 2624 slices in the training dataset. The loss function hyperparameters $\lambda ,$ $\beta ,$ ${\mathrm{\gamma }}$ and ${\mathrm{\delta }}$ are set as 10, 5, 20, 5, respectively. In the original CycleGAN paper (Zhu *et al* 2017), certain weights were assigned to these loss components, including the adversarial (1), cycle consistency (10), identity (5) loss. The optimal weight for the supervised loss (${\mathrm{\gamma }},{\mathrm{and}}\,{\mathrm{\delta }}$) are found through hyperparameter tunning. The learning rate for Adam optimizer is set as 2e-4, and the networks are trained on an NVIDIA Geforce 4090 GPU with 24 GB of memory with a batch size of 1. During training, 4.2 GB CPU memory and 7 GB GPU memory were used for each batch optimization. Each iteration took approximately 2 min during the training phase. The training process was terminated after 600 iterations, with the training of each model taking approximately 30 h in total. In the testing phase, generating a LET_d_ map for a single patient required about 20 s.

For the evaluation of the proposed CycleGAN and two reference models, we utilized N-fold cross validation. In this evaluation technique, data from nine patients was omitted from each dataset (both training and testing) during the training phase. The remaining data was then used as the testing dataset. This process was carried out three times, with patients randomly selected each time for this purpose.

For quantitative comparisons, synthetic LET_d_ maps are compared with ground truth LET_d_ maps using the mean absolute error (MAE), peak signal-to-noise ratio (PSNR) and normalized cross correlation (NCC). MAE quantifies the average pixel-wise difference between synthetic and ground-truth images. However, MAE is not adept at capturing perceptual discrepancies, making it sometimes inadequate for a holistic assessment of synthetic image quality. Thus, metrics like NCC and PSNR are often paired with MAE for a more nuanced evaluation. PSNR, predominantly used in image compression and restoration, gauges the quality of reconstructed images *vis-à-vis* their originals. Its simplicity has made it popular, yet its reliance on mathematical closeness sometimes misaligns with human visual perception. For instance, images with a high PSNR might visually differ, while those with a lower PSNR might appear perceptually identical. To mitigate PSNR’s perceptual limitations, metrics like NCC have found utility in synthetic image assessments. NCC, a similarity metric, is particularly suited for gauging perceptual and structural resemblances, offering a complementary perspective to MAE and PSNR in evaluating synthetic imagery.

MAE measures the magnitude of the difference between the generated image and the ground truth image, as shown in equation (14). Where $f\left(i,j\right)$ is the value of pixel $\left(i,j\right)$ in the ground truth image, $t(i,j)$ is the value of pixel $\left(i,j\right)$ in generated image, and ${n}_{x}{n}_{y}$ are the number of voxels, ${{\mathrm{n}}}_{{\mathrm{z}}}$ is the number of slices

\begin{eqnarray*}\mathrm{MAE}=\frac{1}{{n}_{x}{n}_{y}{n}_{z}}\displaystyle \sum _{i,j,k}^{{n}_{x}{n}_{y}{n}_{z}}\left|f\left(i,j\right)-t(i,j)\right|.\end{eqnarray*}

Peak signal-to-noise ratio (PSNR) is an engineering term for the ratio between the maximum signal and the noise. PSNR is calculated with equation (15). Where *MAX* is the maximum signal intensity possible and *MSE* is the mean-squared error of the image.

\begin{eqnarray*}\mathrm{PSNR}=10\times {\mathrm{log}}_{10}\Space{0ex}{1.0ex}{0ex}(\frac{{\mathrm{MAX}}^{2}}{\mathrm{MSE}}\Space{0ex}{1.0ex}{0ex}).\end{eqnarray*}

The NCC is a measure of the similarity of image structures and is widely used in pattern matching and image analysis (Yoo and Han 2009). NCC is calculated with equation (16). Where ${\sigma }_{{\mathrm{f}}}{\sigma }_{{\mathrm{t}}}$ are the standard deviation of the ground truth and generated images

\begin{eqnarray*}\mathrm{NCC}=\frac{1}{{n}_{x}{n}_{y}}\displaystyle \sum _{i,j}^{{n}_{x}{n}_{y}}\frac{1}{{\sigma }_{f}{\sigma }_{t}}\left|f\left(i,j\right)\times t(i,j)\right|.\end{eqnarray*}

To illustrate the statistical significance of quantitative improvement by the proposed CycleGAN, paired two-tailed t-tests (*α* = 0.05) were used for comparison of the outcomes between numerical results groups calculated from 9 patients’ data in test dataset. The two-tail paired t-test can validate whether the improvement of the proposed model is significant or not compared with other reference models.

All doses and anatomical contours related to the dataset were derived from clinically approved treatment planning data. These contours were exported to MATLAB to carry out the LET_d_ volume metrics calculation on specific ROIs, further evaluating the performance of the proposed methods in clinical practice. The mean value comparisons and LET_d_ volume histograms calculations at 95%, 50%,10% and 5% were performed for the CTV, rectum, and bladder. For the left and right femoral heads (Fem_Head_L and Fem_Head_R), only the mean values were compared.

## 3. Results

### 3.1. Image synthetic generation performance comparison

**Figure 4.**
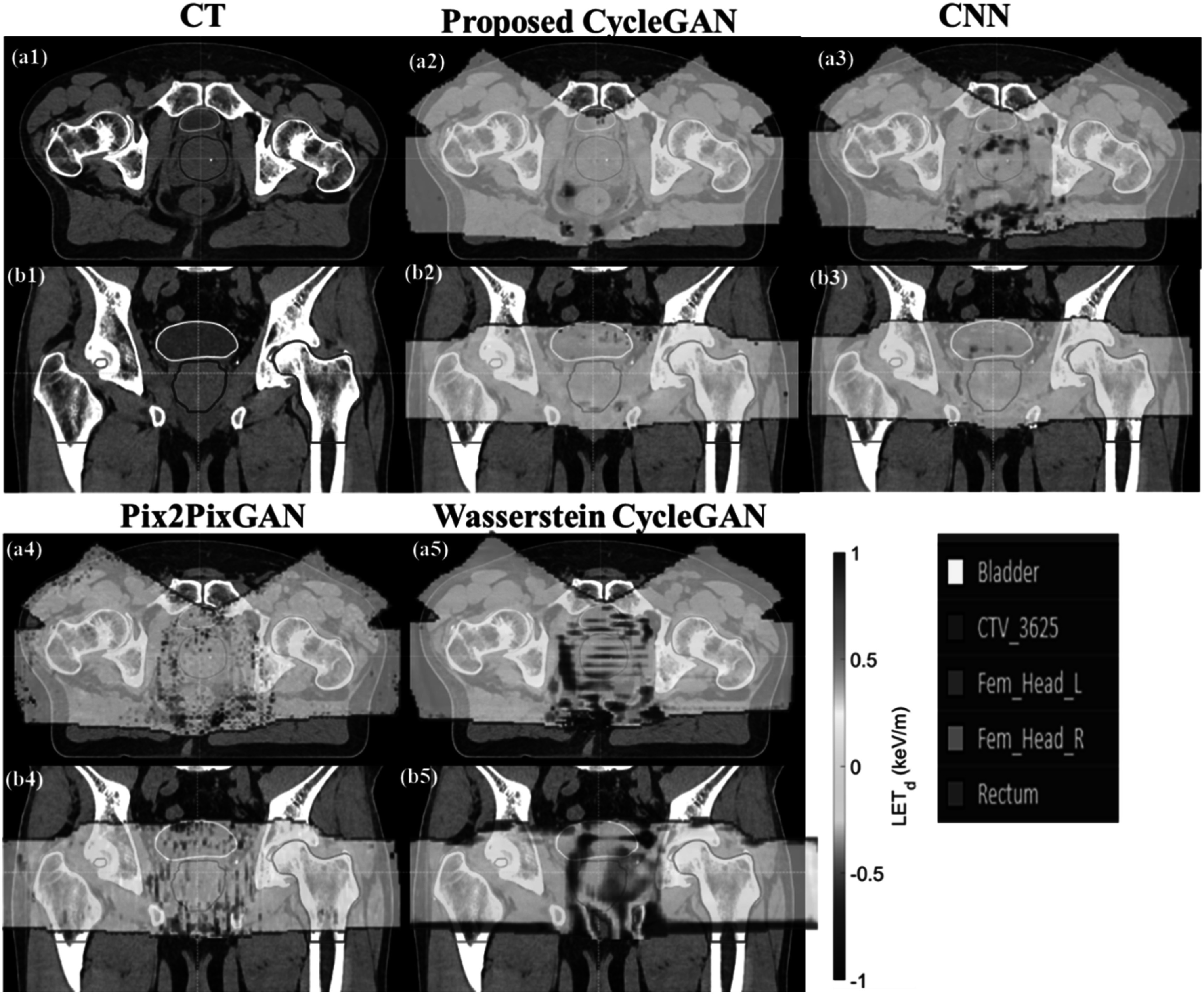
Patient’s CT (a1–b1) with contour information and cross-section view of comparison of estimation error map produced by proposed CycleGAN (a2–b2). CNN (a3–b3), pix2pixGAN (a4–b4), Wasserstein CycleGAN (a5–b5), The regions-of-interest (ROIs) shown in the figure include CTV, Rectum, Bladder, left femur head (Fem_Head_L) and right femur head (Fem_Head_R).

Table 1 summarizes the numerical results of various methods applied to the generation of synthetic LET_d_ maps. As shown in table, the proposed CycleGAN surpasses the performance of the other three models in terms of MAE, PSNR, and NCC. The pix2pixGAN demonstrates the second-best performance in terms of MAE, PSNR and NCC, whereas the performance of the Wasserstein CycleGAN is the least impressive among the methods examined. It should be noted that the calculation of MAE was only performed on voxel data points within a mask (with a dose threshold of 50 cGy_RBE_), implying that the image background was not incorporated in the MAE computation. The performance of CNN is comparable to that of pix2pixGAN, though it is slightly inferior. The proposed CycleGAN demonstrates a MAE of 0.096 keV *μ*m^−1^, a value substantially smaller (approximately 3%–5%) than the typical LET_d_ value at each voxel (around 2–3 keV *μ*m^−1^, shown in figure 4). Additionally, the proposed CycleGAN exhibits a lower standard deviation compared to the other models in terms of MAE, PSNR and NCC.

**Table 1.** Numerical results of different methods on pelvic sLET_d_ image.

	CNN	Pix2Pix GAN	Wasserstein cycleGAN	Proposed cycleGAN
MAE (keV/*μ*m)↓	0.283 ± 0.132	0.242 ± 0.081	0.347 ± 0.037	**0.096** ± **0.019**
PSNR (dB)↑	16.596 ± 6.963	17.817 ± 2.805	9.651 ± 5.973	**24.203** ± **2.683**
NCC↑	0.962 ± 0.005	0.980 ± 0.002	0.829 ± 0.009	**0.997** ± **0.002**

Table 2 displays the results of the two-tailed paired t-test comparing the proposed CycleGAN with the other three reference models. The results demonstrate that the proposed CycleGAN significantly outperforms the other two reference models in terms of MAE, PSNR and NCC.

**Table 2.** P values by performing a two-sided t-test (*α* = 0.05) between proposed method and comparison methods for MAE and PSNR on pelvic sLETd maps.

	CNN versus proposed CycleGAN	Pix2Pix GAN versus proposed CycleGAN	Wasserstein CycleGAN versus proposed CycleGAN
MAE (keV/*μ*m)	<0.001	0.003	<0.001
PSNR (dB)	<0.001	<0.001	<0.001
NCC	<0.001	<0.001	<0.001

Figure 4 provides a visual representation of the estimation errors generated by the reference models and our proposed CycleGAN network. The proposed CycleGAN outperforms the other three reference models. The Pix2PixGAN tends to overestimate the LET_d_ value at bladder, CTV, Fem_Head_L and Fem_Head_R, while underestimating LET_d_ at the rectum. Figures 4(a3–b3) and (a5–b5) depicts the estimation errors generated by CNN and Wasserstein CycleGAN. The visualizations indicate that this model exhibits the poorest performance among all four methods examined.

**Figure 5.**
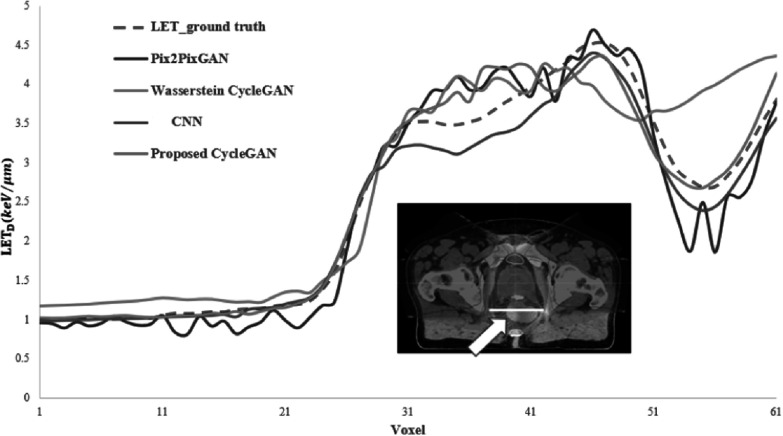
Comparison of LET_d_ values along profiles indicated by white lines in LET_d_ map, the ground truth LET_d_, and estimated by CNN, pix2pixGAN, Wasserstein CycleGAN, and proposed Cycle GAN are shown.

Figure 5 presents the comparison of LET_d_ values along a profile line passing through the rectum of a patient, as predicted by the four DL models: CNN, Pix2PixGAN, Wasserstein CycleGAN, and our proposed CycleGAN. The proposed method aligns with the ground truth better than the other two reference models. The Wasserstein CycleGAN significantly overestimates the LET_d_ value from voxel 51 to 61, whereas the Pix2pixGAN underestimates the LET_d_ value from voxel 53 to 60. CNN exhibits a mismatch with the ground truth data and appears to consistently underestimate the LET_d_ values for voxels ranging from 28 to 41.

**Figure 6.**
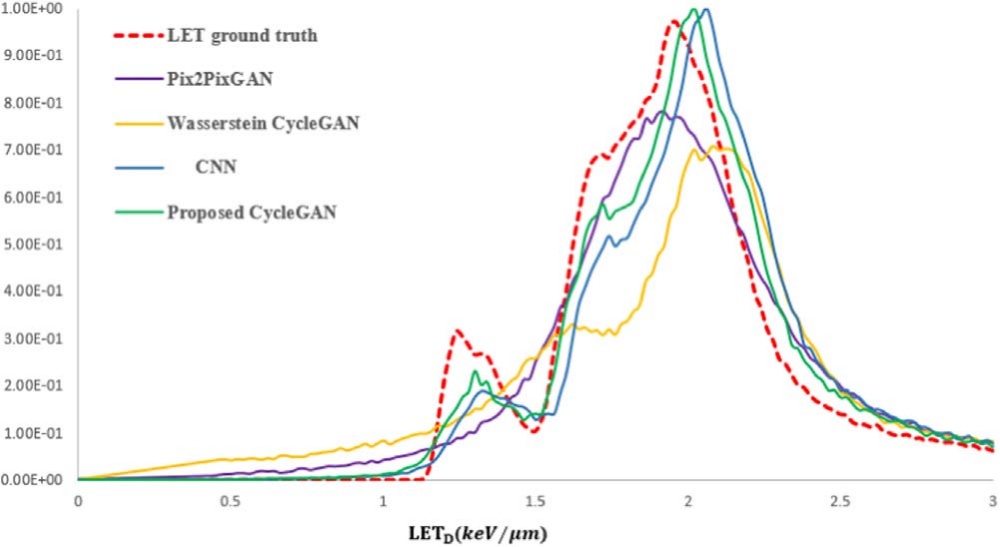
Comparison of histogram of LET_d_ map ground truth and synthetic LET_d_ map generated by, CNN pix2pixGAN, Wasserstein CycleGAN, and Proposed CycleGAN.

Figure 6 shows the synthetic LET_d_ histograms generated by the proposed CycleGAN, CNN, Pix2pixGAN, and Wasserstein CycleGAN, along with the ground truth for comparison. As shown in figure 6, the proposed CycleGAN demonstrates a better match in terms of LET_d_ value distribution when compared to the reference models.

### 3.2. Quantitative analysis in clinical practice

**Figure 7.**
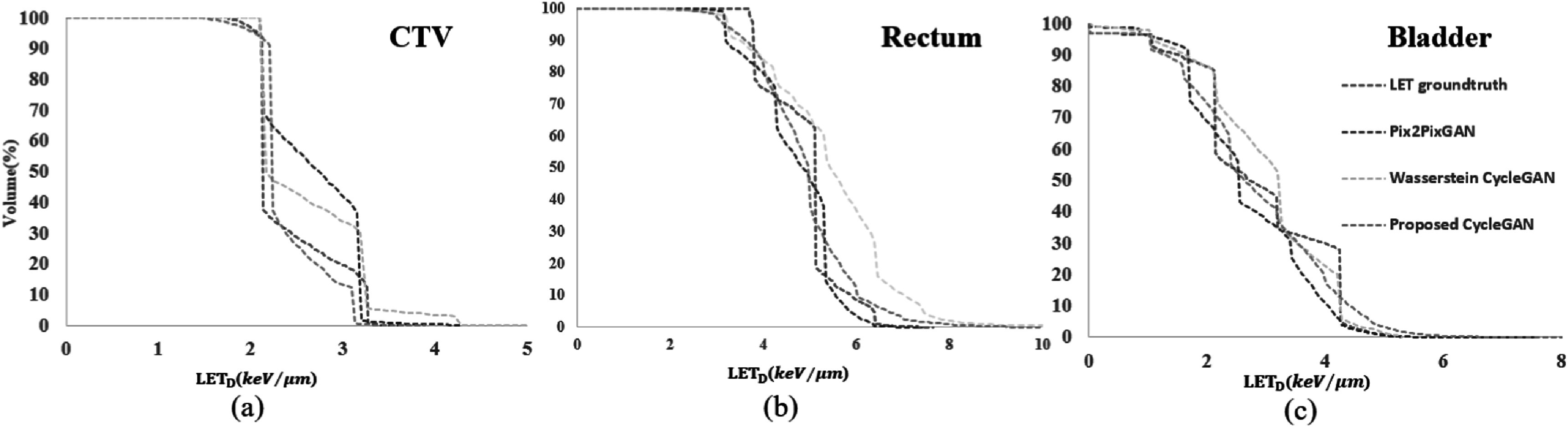
Comparison of LET_d_-volume histograms (DLVHs) for a single treatment plan, comparing the ground truth with the Pix2pixGAN, Wasserstein CycleGAN, and the proposed CycleGAN.

The LET_d_-volume metrics and mean values for the CTV and specific OARs, including the rectum, bladder, Fem_Head_L, and Fem_Head_R, are detailed in proposed CycleGAN exhibited the best performance for small volume metrics at the bladder, it was outpaced by the Wasserstein CycleGAN and Pix2pixGAN at the CTV and rectum. CNN is not evaluated in this study. Figure 7 shows the comparison of dose-linear energy transfer histogram (DLVH) for a single treatment plan, comparing the ground truth with the Pix2pixGAN, Wasserstein CycleGAN and proposed CycleGAN.

As shown in table 3, the proposed CycleGAN demonstrated superior performance in terms of large and median volume metrics at CTV, rectum and bladder, with the exception of the CTV-L_50%._ Though the proposed CycleGAN exhibited the best performance for small volume metrics at the bladder, it was outpaced by the Wasserstein CycleGAN and Pix2pixGAN at the CTV and rectum. The performance of our proposed method aligns closely with that of the reference models when evaluated at CTV-L_10%_ and CTV-L_5%._ For the metric Rectum-L_10%_, our proposed method surpasses the Wasserstein CycleGAN in performance. It aligns closely with the performance of pix2pix GAN but exhibits a reduced standard deviation. In evaluations using low-volume metrics, the relatively few data points may introduce errors, potentially obscuring the superior performance of our proposed method.

**Table 3.** The mean LET_d_ error and LET_d_-volume metrics, computed by the proposed CycleGAN, Pix2pixGAN, and Wasserstein CycleGAN at CTV and specific OARs, including the Rectum, Bladder, Fem_Head_L, *and* Fem_Head_R, are provided in terms of keV/*μ*m.

	Pix2PixGAN (keV/*μ*m)	Wasserstein cycleGAN (keV/*μ*m)	Proposed cycleGAN (keV/*μ*m)
CTV-Mean	0.287 ± 0.132	**0.197** ± **0.125**	0.213 ± 0.103
CTV-L_95%_	0.052 ± 0.032	0.119 ± 0.112	**0.031** ± **0.012**
CTV-L_50%_	0.580 ± 0.272	**0.038** ± **0.042**	0.077 ± 0.021
CTV-L_10%_	0.103 ± 0.053	**0.067** ± **0.078**	0.128 ± 0.035
CTV-L_5%_	**0.104** ± **0.056**	0.150 ± 0.089	0.118 ± 0.024
CTV-L_1%_	**0.143** ± **0.065**	1.001 ± 0.825	0.147 ± 0.035
Rectum-Mean	0.621 ± 0.356	1.388 ± 1.235	**0.211** ± **0.086**
Rectum-L_95%_	0.032 ± 0.016	0.074 ± 0.085	**0.024** ± **0.013**
Rectum-L_50%_	0.630 ± 0.578	1.115 ± 0.623	**0.612** ± **0.133**
Rectum-L_10%_	**0.570** ± **0.465**	2.083 ± 1.854	0.626 ± 0.231
Rectum-L_5%_	0.478 ± 0.293	1.082 ± 1.134	**0.272** ± **0.095**
Rectum-L_1%_	**1.274** ± **1.125**	2.234 ± 1.895	1.536 ± 0.968
Bladder-Mean	0.462 ± 0.256	**0.202** ± **0.113**	0.277 ± 0.112
Bladder-L_95%_	0.141 ± 0.075	0.073 ± 0.089	**0.035** ± **0.027**
Bladder-L_50%_	0.177 ± 0.102	0.485 ± 0.359	**0.101** ± **0.081**
Bladder- L_10%_	0.371 ± 0.256	0.193 ± 0.187	**0.179** ± **0.096**
Bladder- L_5%_	0.521 ± 0.358	**0.326** ± **0.273**	0.401 ± 0.157
Bladder- L_1%_	0.891 ± 0.678	0.992 ± 0.965	**0.602** ± **0.257**
Fem_Head_L-Mean	**0.001** ± **0.001**	0.083 ± 0.032	0.018 ± 0.004
Fem_Head_R-Mean	**0.003** ± **0.002**	0.045 ± 0.042	0.013 ± 0.003

## 4. Discussion

There’s a growing body of evidence suggesting that LET_d_ values could be utilized in the biological optimization of treatment plans to enhance the therapeutic ratio in proton therapy (Grassberger and Paganetti 2011, Giantsoudi *et al* 2013). In this work, we present a framework that utilizes a paired CycleGAN to generate synthetic LET_d_ maps from dose maps. The work provides a detailed account of the customized paired CycleGAN structure and the specific hyperparameters employed. The proposed CycleGAN demonstrates superior performance in image synthesis evaluations, as evidenced by its better MAE, PSNR, and NCC results, compared to those of the Pix2pixGAN and Wasserstein CycleGAN.

Our work stands out as, to the best of our knowledge, it represents the first instance of using a paired CycleGAN for predicting LET_d_ from treatment dose plans in proton radiation therapy for prostate cancer. The MAE of the proposed CycleGAN is 0.096 ± 0.019 keV *μ*m^−1^, which is approximately 5% of the most typical LET_d_ value in our dataset (as shown in figure 4). This is also around 4%, 3%, and 2% of the mean LET_d_ value at CTV (mean value is 2.42 keV *μ*m^−1^), rectum (mean value is 3.21 keV *μ*m^−1^), and bladder (mean value is 4.24 keV *μ*m^−1^), respectively. Though we are the first to publish results on proton therapy for prostate cancer using a paired CycleGAN, we can compare its performance with other methods for predicting LET_d_ across various anatomic sites. Pirlepesov *et al* developed an Artificial Neural Network (ANN) to predict the LET_d_ map for cranial patients undergoing proton therapy (Pirlepesov *et al* 2022). The Root Mean Square (RMS) difference reported in their study ranges from 0.5 to 1 keV *μ*m^−1^ for various OARs, whereas our proposed method achieved an RMSE of 0.57 keV *μ*m^−1^ at prostate. Before the adoption of DL methods, analytical approaches were the practical options for calculating LET_d_ to avoid the significant time and effort required for Monte Carlo simulations. Wilkens *et al* developed an analytical method to calculate LET_d_ along the central axis of broad beams in water, with an observed maximum deviation of 0.5 keV *μ*m^−1^ (Wilkens and Oelfke 2003). Marsolat *et al* suggested a correction factor for Wilkens’ model, thereby improving the mean LETd deviation along the beam axis to 0.05 keV *μ*m^−1^ (Marsolat *et al* 2016). It is important to note that these methods perform LET_d_ calculations for a proton beam in water, a scenario significantly less complex in terms of structure and physical conditions compared to the prostate cancer patients undergoing SBRT treatments presented in this work.

LET_d_ is a physical quantity that is difficult to measure or calculate accurately. At present, MC simulations are the golden standard for LET_d_ calculation. However, implementing the MC method for individual patient LET_d_ calculation involves considerable effort. This includes but is not limited to reproducing complex geometrical structures and accommodating complicated physical conditions. Various patient body sizes and the relative positioning of patients during treatment make the reproduction of precise geometry in MC software a significant challenge. Choosing the correct physical conditions for Monte Carlo simulations also poses complexity to the process. The physics involved are quite intricate; for instance, incorporating secondary protons into the simulation can alter the results compared to considering only primary protons (Kalholm *et al* 2021). Beyond the efforts in implementing the MC algorithm, the computational cost is another significant challenge. Most MC codes used for calculating LET_d_, such as Geant4-TOPAS (Polster *et al* 2015), are still CPU-based, which makes the simulation quite time-consuming (more than several minutes). The proposed framework addresses these challenges. Once the DL network is trained, the only necessary input is the dose plan map. From this, the network can generate a highly accurate LET_d_ map in less than 20 s. This significant reduction in time and computational resources could greatly enhance the efficiency and applicability of proton therapy treatment planning.

In the treatment planning process of prostate cancer proton therapy, two OARs, the rectum and the bladder, are likely to present a challenge. However, the proposed method demonstrates good performance on large, medium, and small volume metrics for these OARs and the CTV, with differences smaller than 0.5 keV *μ*m^−1^. A notable exception is at the small volume metrics of the rectum where the observed difference is 0.726 keV *μ*m^−1^. This is likely due to the smaller number of data points in these metrics and the fact that the rectum itself has a higher LET_d_ value. The maximum observed differences in the mean LET_d_ value across all examined OARs and the CTV are less than 0.3 keV *μ*m^−1^, which means the synthetic LET_d_ map has a good agreement with the ground truth.

Despite the promising performance of the proposed method in generating synthetic LET_d_ maps, there are still limitations in this work. The accuracy of our proposed method is still contingent upon the quality of the ground truth, which is the LET_d_ map generated using the vendor’s internal MC code. Moreover, the trained network did not exhibit robustness across different optimization strategies. The optimization strategy of the training dataset used SFO. However, when we evaluated our trained network with a test dataset that employed a multi-field optimization (MFO) strategy, the MAE increased to 0.6 keV *μ*m^−1^. Additionally, we observed that the mean difference in the OARs and CTV exceeded 0.5 keV *μ*m^−1^. Moving forward, we aim to enrich our proposed framework by incorporating a more extensive training dataset to enhance the performance and robustness of this approach.

KBP approaches in proton therapy hold the promise of delivering the advantages observed with their application in photon treatment planning. These benefits encompass enhanced plan quality and consistency (Li *et al* 2017), improved treatment planning workflows (Good *et al* 2013, Ge and Wu 2019). The advantages in proton therapy are further amplified, enabling a comparative analysis of plans between proton and traditional photon-based radiation therapy. This aids in identifying patients who are most likely to benefit from proton therapy. Our introduced framework, capable of delivering precise LET_d_ distribution data, holds promise in integrating the KBP approach within the realm of LET_d_. Given that the LET_d_ of protons rises at the distal end and there exists a robust correlation between RBE and LET_d_ value, LET_d_ is deemed a pivotal determinant in assessing potential variable RBE within proton therapy. LET-based optimization can ensure that critical structures are spared from this heightened biological damage. A review of the literature reveals promising indications for the broad adoption of LET-based optimization (Deng *et al* 2021). This optimization is contingent upon rapid and precise LET_d_ distribution data, which our proposed methodology is equipped to furnish. Moreover, should we obtain the ground truth for the LET-based optimization plan, it would be feasible to adapt our proposed framework to synthesize the LET-based optimization plan derived from the original plan.

While the paired CycleGAN has shown commendable performance in this study, several recently developed DL models have reportedly outperformed the CycleGAN in image synthesis tasks (Ho *et al* 2020, Sano *et al* 2021), such as the diffusion denoising probabilistic model. We are motivated to incorporate more advanced DL models to further enhance the accuracy of LET_d_ prediction in the future. The LET_d_ map information also plays a crucial role in other anatomical sites such as head-and-neck and breast. Moving forward, it will be necessary to train the proposed model with datasets from various anatomical sites. The proposed method operates on 2D slices and does not employ a patch extraction strategy. It is conceivable that 3D patch-based models might offer improved structural preservation and more effectively capture spatial relationships across multiple dimensions. The proposed method could potentially be extended to generate LET_d_ maps for other treatment modalities, such as Carbon-ion therapy, which is also a topic of future work.

## 5. Conclusion

We proposed a framework utilizing paired CycleGAN to generate synthetic LETd maps for prostate cancer patients undergoing SBRT treatment, derived directly from the treatment dose plans. The accuracy of the proposed method was scrutinized through quantitative evaluation and compared with two other reference DL models. The method we’ve proposed demonstrates high accuracy and efficiency in predicting LET_d_ maps. This framework has the potential to significantly benefit more proton therapy centers by improving treatment plan quality through RBE optimization, aided by the provision of highly accurate LET_d_ information.

## Data Availability

The data cannot be made publicly available upon publication because the cost of preparing, depositing and hosting the data would be prohibitive within the terms of this research project. The data that support the findings of this study are available upon reasonable request from the authors.
